# Metabolite Profiling of *Justicia gendarussa* Burm. f. Leaves Using UPLC-UHR-QTOF-MS

**DOI:** 10.3797/scipharm.1511-08

**Published:** 2015-04-14

**Authors:** Indah Yulia Ningsih, Diah Intan Purwanti, Suwidji Wongso, Bambang E. W. Prajogo, Gunawan Indrayanto

**Affiliations:** 1 Faculty of Pharmacy, Jember University, Jl. Kalimantan I, 68121, Jember, Indonesia; 2 PT. Angler BioChemLab, Plaza Graha Family C-25, 60226, Surabaya, Indonesia; 3 Faculty of Pharmacy, Airlangga University, Jl. Dharmawangsa Dalam, 60286, Surabaya, Indonesia

**Keywords:** *Justicia gendarussa*, UPLC-UHR-QTOF-MS, PCA, HCA, Sites of cultivation, Significant metabolites

## Abstract

An ultra-performance liquid chromatography ultra-high-resolution quadrupole-time-of-flight-mass spectrometry (UPLC-UHR-QTOF-MS) metabolite profiling ofxs *Justicia gendarussa* Burm. f. leaves was performed. PCA and HCA analyses were applied to observe the clustering patterns and inter-sample relationships. It seemed that the concentrations of Ca, P, and Cu in the soil could affect the metabolite profiles of *Justicia gendarussa*. Six significant metabolites were proposed.

## Introduction

Gandarusa (*Justicia gendarussa* Burm. f.) can be found wild or cultivated in Indonesia, India, China, Malaysia, Sri Lanka, the Philippines, and Bangladesh. The leaves of this plant have been reported for anti-angiogenic [1], antioxidant [2], antibactericidal [3], antifungal [4], anti-arthritic [5], anti-inflammatory and antinociceptive [6], antisickling [7], anthelmintic [8], cytotoxicity [9], larvicidal, and adulticidal activities [10]. In addition, its aerial parts showed *in vitro* HIV type 1 reverse transcriptase inhibitor [11], anti-inflammatory and analgesic [12], sedative, and hypnotic activities [13]. This plant has also been used by people in Papua as a male contraceptive [14]. *In vitro* and *in vivo* antifertility tests of *n*-butanol fractions of *J. gendarussa* showed that the main mechanism was through competitive and reversible inhibition of spermatozoa hyaluronidase enzyme [15].

*J. gendarussa* leaves obtained from Pacet, Indonesia contained 6,8-di-*C-α-L*-arabinosyl-apigenin, 6-*C-α-L*-arabinosyl-8-*C-β-D*-xylosyl-apigenin [15], and justidrusamides A-D [16]. Whereas the species collected from India contained *β*-sitosterol, friedelin, lupenol [17], and *O*-disubstituted aromatic amines (2-amino-*O*-methyl-benzyl alcohol, 2-(2’-amino-benzylamino)-*O*-methyl-benzyl alcohol, 2-amino-benzyl alcohol, 2-(2’-amino-benzylamino)-benzyl alcohol) [18]. Furthermore, phytochemical screening also showed the presence of vitexin [19].

Environmental factors, such as the site of cultivation, altitude, temperature, sun exposure time, rainfall, climate, and soil can influence the primary and secondary metabolites of plants [20–22]. These factors may affect secondary metabolites qualitatively and quantitatively, so their bioactivities could be varied [21–23]. Therefore, metabolite profiling studies of herbal plants is very important for ensuring their safety and efficacy.

LC-DAD-APCI-MS-based metabolite profiling of three species of *Justicia*, namely *J. secunda*, *J. graciliflora*, and *J. refractifolia*, had been reported [24]. To the best of our knowledge, there is no report written on any metabolite profiling of *J. gendarussa* originating from Indonesia before. In this work, UPLC-UHR-QTOF-MS was used to determine metabolite profiles of *J. gendarussa* leaves from different sites of cultivation (Table 1).

**Tab. 1:**
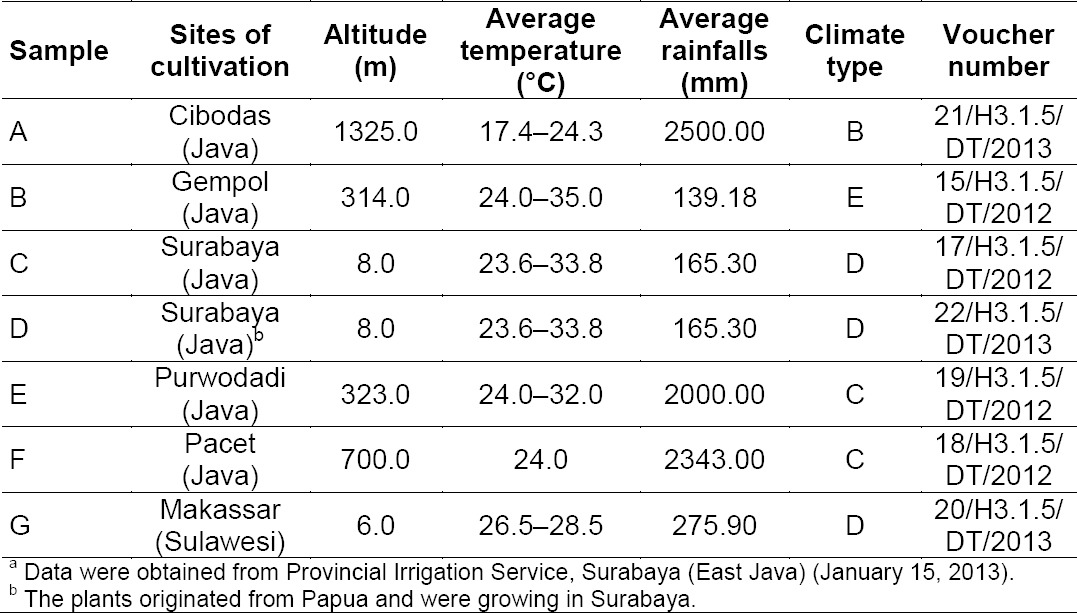
Origin of samples^a^

## Results and Discussion

**Fig. 1:**
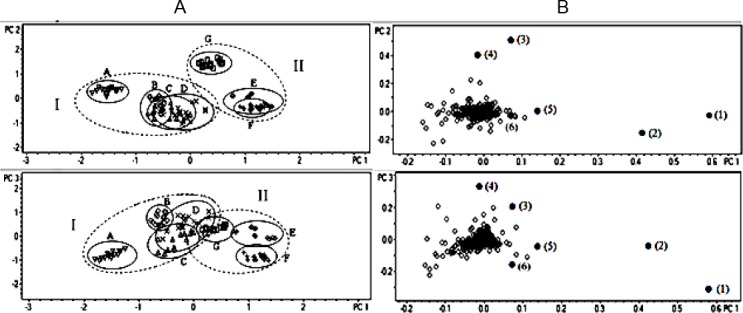
PCA score plot (A) and loading plot (B); explained variants PC1: 33.3%, PC2: 17.40%, and PC: 13.60%. Numbers (1–6) refer to significant metabolites as listed in Table 2

**Fig. 2:**
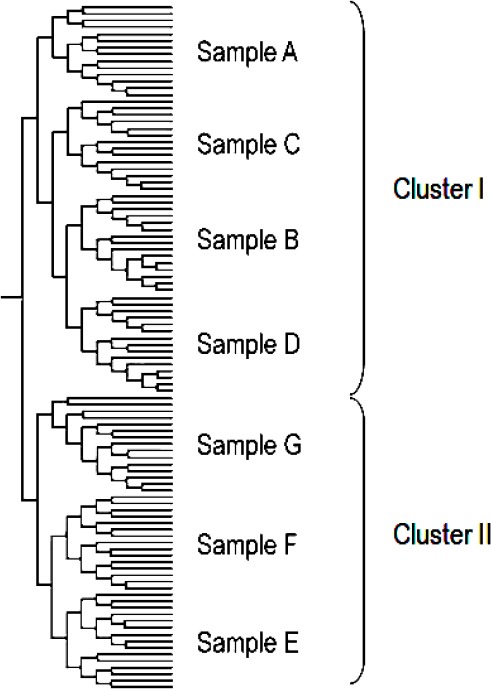
Dendogram of *J. gendarussa* leaves extract

PCA analysis of pair RT and *m/z* (Figure 1A) showed a definite discrimination of samples A, E, F, and G, whilst samples of B, C, and D were not well separated. The total variants explained by the three principle components (PC1, PC2, PC3) was 64.3%. In order to confirm the clusters observed in PCA, HCA analysis was also performed. A dendogram of all the samples (Figure 2) indicated that samples were comprised of two clusters; cluster I consisted of samples (A, B, C, D), and cluster II was (E, F, G).

The location of B was very close to C and D. Samples C and D were cultivated on the same location, but their plants’ origins and soil types were different.

**Fig. 3:**
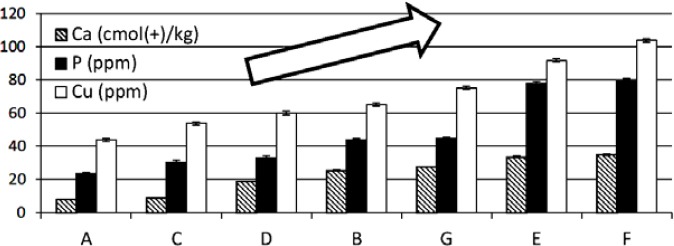
Concentrations of calcium, phosphorus, and copper in soils

Two-way ANOVA showed significant differences between the concentrations of Ca, P, Cu, K, and Fe (p < 0.05) in soils at the sites of cultivation. Figure 3 showed significant positive correlation trends of the concentrations of Ca, P, and Cu in soils versus the metabolite profile’s clustering of samples A, C, D, B, G, E, F (correlation coefficients were 0.771, 0.624, and 0.759, respectively; r table was 0.549 for p = 0.01). On the contrary, concentrations of Fe and K (data not shown) in soils did not yield any correlations with metabolite profiles of all samples (r = −0.382, and 0.041, respectively). It seemed that concentrations of Ca, P, and Cu could affect the metabolite profile of the samples, whilst concentrations of Fe and K in soils, altitude, average temperature, average rainfall, and climate type (Table 1) could not affect the profiles of the metabolites. PCA showed that samples B, C, and D could not be well-separated (Figure 1A), although the concentrations of Ca, P, Cu, K, and Fe in the soils were significantly different (as described above). These indicated that other elements of the soils that were not determined in this work might also affect the metabolite profiles. Freitas *et al*. [25] reported the influence of soil nutrients (N, P, K, Ca, and B) on the secondary metabolite production in *Passiflora alata*.

### Identification of Significant Metabolites

**Fig. 4:**
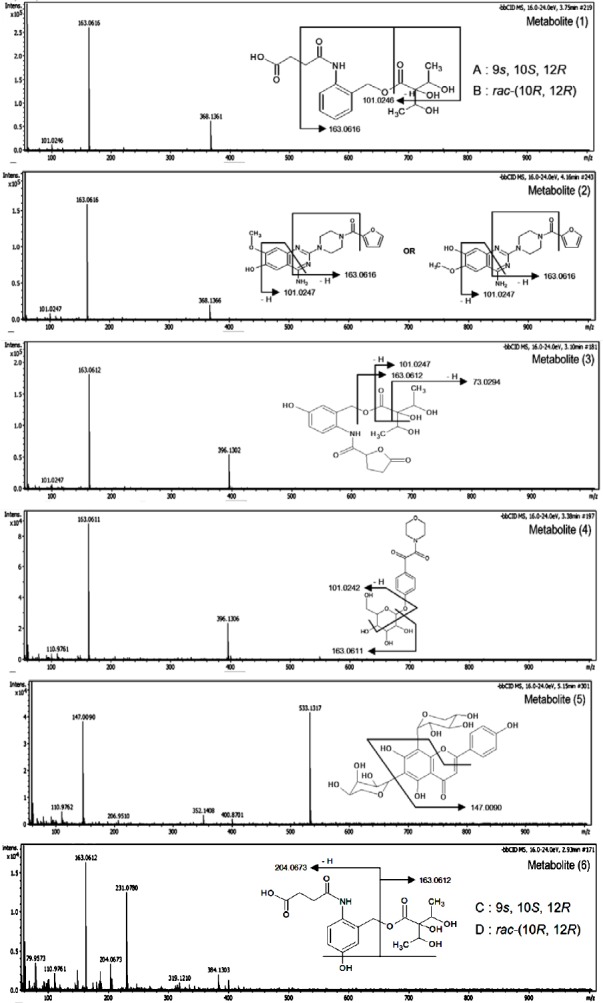
Chemical structures of the proposed compounds and their fragmentation

Six significant metabolites (1–6) that mainly contributed to the grouping of the samples were presented in the loading plots (Figure 1B). Metabolite identification was performed using several software programs of the instrument (accurate mass of parent ion, isotopic pattern, and fragmentation pattern of the compounds; see “Experimental”), and by comparison to data available in databases (METLIN [26], Chemspider [27], and PubMed [28]). The proposed compounds and their mass fragmentation patterns are shown in Tables 2 & 3, and Figure 4. Metabolites (1), (5), and (6) were previously reported for *J. gendarussa* leaves [15, 16]. Metabolite (2) was reported as a demethylated product of prazosin in liver microsomes for rats, dogs, and humans [29].

**Tab. 2:**
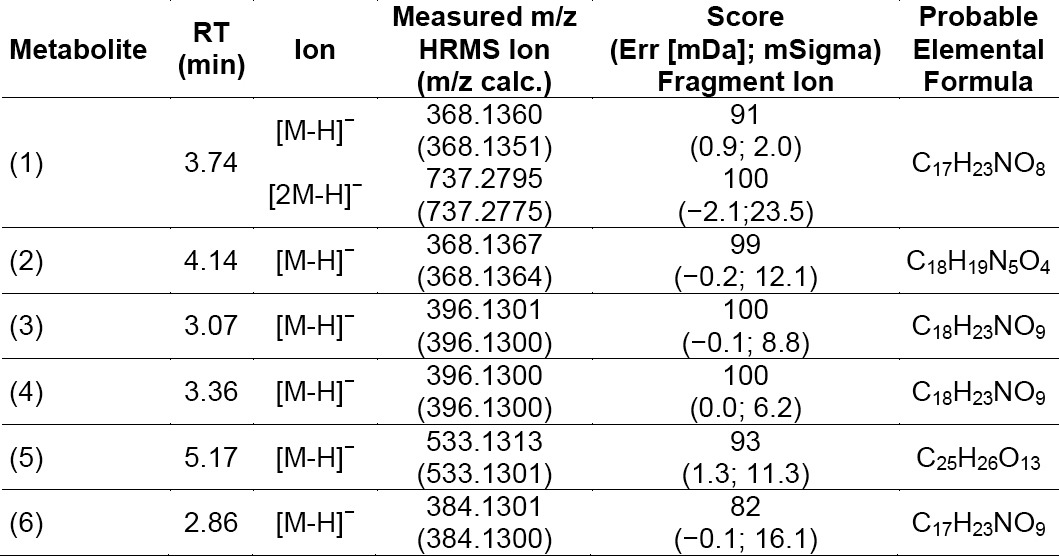
Proposed compounds and its probable elemental formula

**Tab. 3:**
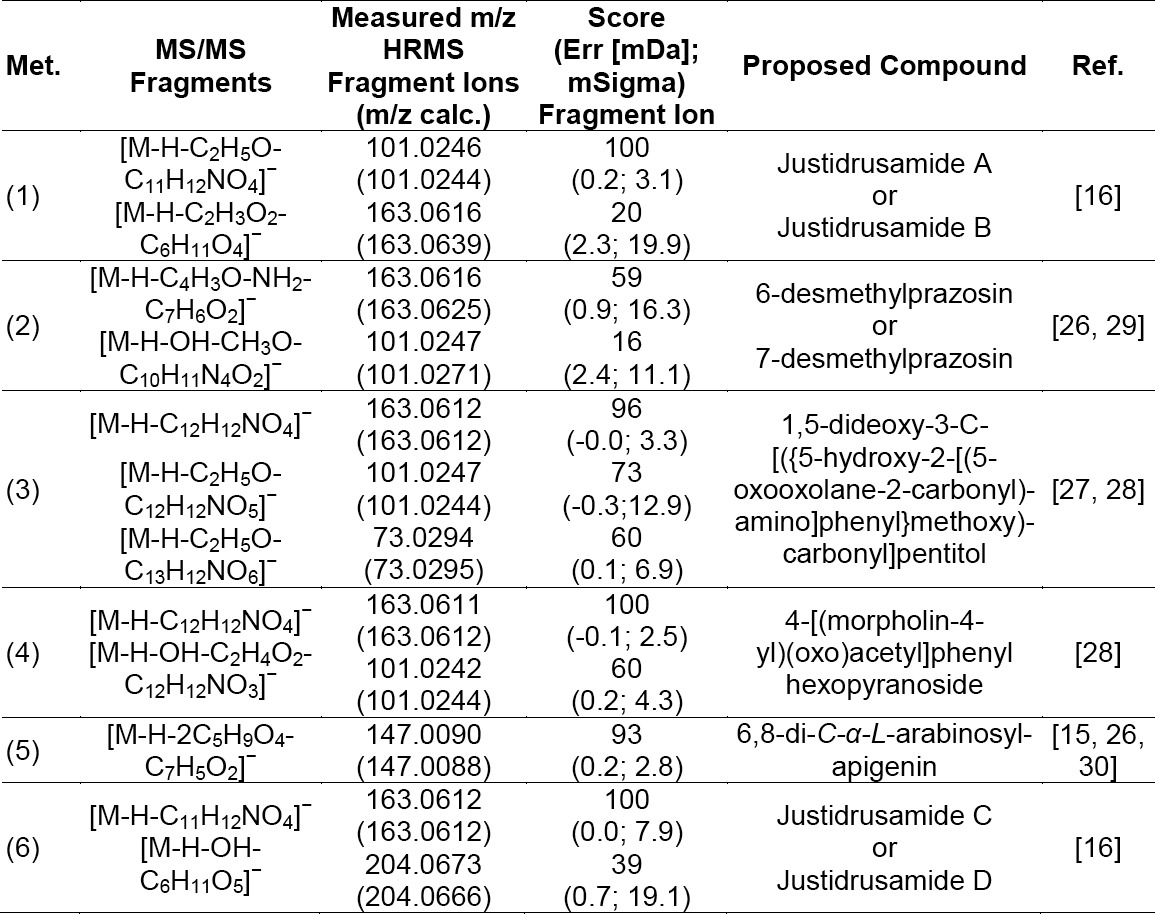
Proposed compounds and its fragmentation

**Fig. 5:**
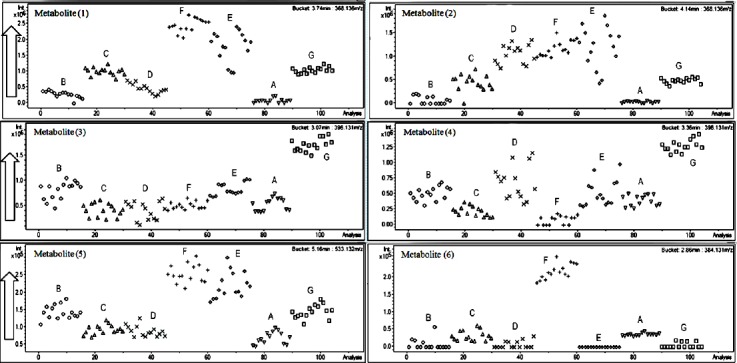
Bucket statistic plots of significant metabolites

Relative intensities of the metabolites are presented in bucket statistic plots (Figure 5); metabolites (1), (2), and (5) were found in relatively high levels in samples E and F, whilst metabolites (3) and (4) were in sample G. The highest intensities of the metabolite (6) was found in sample F.

This study showed that metabolite profiles of *J. gendarussa* were affected by the plant’s cultivation sites. To ensure the quality and efficacy of this medicinal plant, further studies are needed to compare its metabolites and bioactivity profiles.

## Experimental

### Materials and Chemicals

*J. gendarussa* leaves were collected from Pacet, Purwodadi, Surabaya, Gempol, Makassar, and Cibodas between September 2012 to January 2013 (Table 1). Samples were properly authenticated by Department of Pharmacognosy and Phytochemistry, Airlangga University, Surabaya. Mature, dark green leaves of five different plants were collected from each location in triplicate; the leaves were air-dried and powdered. Moisture contents (MC) of the samples were 9.6 ± 1.7%, n = 105 (by using Moisture Analyzer HB43-S, Mettler Toledo). The maximum permitted level of MC of the herbal medicine was 12%, w/w [31].

Soil collection was performed by using composite sampling. Fifteen sub-samples were collected randomly 6–8 inches from the surface [32].

Methanol, 2-propanol, and formic acid were analytical reagents from Merck (Darmstadt, Germany). Purified water was from Sigma-Aldrich (St. Louis, MO, USA), acetic acid from J.T. Baker (Phillipsburg, NJ, USA), and NaOH from Agilent (Agilent solution for HPCE). All samples were filtered through a 0.2 µm Agilent Econofilter PVDF 13m.

### Preparation of Extracts

Two ml of methanol containing 0.1% formic acid was added to 20.0 mg of dried leaf powder. The samples were vortexed for 15 s, sonicated for 2 min, then vortexed again for 2 min, and followed by centrifugation at 13,000 *x g* for 10 min. The extraction process was repeated three times. Supernatants were collected and dried using N_2_. Two ml of 50% methanol was added to the residues and vortexed for 1 min before injection into the UPLC.

QC samples were prepared by mixing all of the samples equally that were measured in a series of experiments.

### Instrumentation

Samples were analyzed using an UPLC Dionex Ultimate 3000 RS LC coupled to QTOF Bruker Maxis Impact HD (Bruker Daltonics, Bremen, Germany), equipped with an ESI interface operating in negative ion mode, with a mass range of m/z 50-1000, the capillary voltage was 2500 V; dry N_2_ gas flow 8.0 l/min (200°C); nebulizer pressure 2.0 bar; end plate offset 500 V; collision energy 25 eV, and acquisition time factor 1 s.

Chromatographic separation was carried out using an Acclaim RSLC 120 C18 column (2.2 µm, 120 Å, 2.1 x 100 mm) (Dionex). The mobile phase consisted of 90% methanol with 5 mM ammonium acetate and 50% methanol with 5 mM ammonium acetate. Injection volume was 1.0 µl. Mass calibration was performed using 1 mM sodium formate/acetate in 50% isopropanol with 0.2% acid; HCOO(NaCOOH)_1_ (m/z 112.9856), Ac(NaAc)_1_ (m/z 141.0169), and Ac(NaF)_1_ (m/z 127.0013).

Data analysis and calculation were performed using the software programs *Data Analysis 4.1* (*SmartFormula, SmartFormula 3D*, and *Fragmentation Explorer*) and *Profile Analysis 2.1* (*PCA, HCA*, and *SmartFormula)* from Bruker Daltonics, Bremen, Germany.

### Soil Analysis

Analyses of Ca, P, Cu, K, and Fe were performed at the Indonesian Spice and Medicinal Crops Research Institute, Bogor, Indonesia. Ca, Cu, K, and Fe analyses were performed using an atomic absorption spectrometer (AAS) at 422.7 and 285.2 nm; 324.7 nm; 766.5 nm; 248.3 nm and 372.0 nm, respectively, in triplicate, whilst P analysis was carried out using a spectrophotometer at 693 nm in triplicate according to the standard methods [33].

### Data Processing

Automatic time alignment was performed on RT-*m/z* pairs of 0.4 to 15 min; data were grouped automatically into buckets with RT-*m/z* pairs of 0.0781 min and m/z 18.6744; the mass range 200-700 Da with mass tolerance 0.05 Da; normalized with the sum of bucket values, *pareto*-scaled and bucket filter 2%. All intensities were corrected to dry weight basis before generating PCA. Hierarchical Clustering Analysis (HCA) was carried out using the Euclidean distance method and Ward linkage method.

The proposed molecular formula was performed using *SmartFormula* based on the exact mass and isotopic pattern; proposed fragmentation of the compound was generated using *SmartFormula 3D*. Then, the fragmentation pattern of the compounds were compared with some databases using *Fragmentation Explorer*.

### Analytical Method Validation

Stability and validation (intraday and interday precisions) were performed by injecting sample C at 0 h, 9 h, 18 h, 27 h, and 36 h in triplicate. PCA analysis confirmed that the extract was stable in 36 h, and showed acceptable intra- and interday precision (data not shown).

For checking the reliability of the method for each series of experiments, the QC sample was injected three times at the beginning of the analysis, then regularly every 8-9 samples. The coefficient variation (CV) of the data set were evaluated according to the published method [34]; our data showed that > 71.50% of the bucket data had CV < 30%. The tight clustering of the QC sample in the PCA analysis showed the reliability of the method (data not shown).
